# Racial and ethnic differences in primary, unscheduled cesarean deliveries among low-risk primiparous women at an academic medical center: a retrospective cohort study

**DOI:** 10.1186/1471-2393-13-168

**Published:** 2013-09-03

**Authors:** Joyce K Edmonds, Revital Yehezkel, Xun Liao, Tiffany A Moore Simas

**Affiliations:** 1 William F. Connell School of Nursing, Boston College, 140 Commonwealth Ave, 02467, Chestnut Hill, MA, USA; 2 Department of Obstetrics and Gynecology, University of Massachusetts Medical School/UMass Memorial Medical Center, 119 Belmont Street, 01605, Worcester, MA, USA

**Keywords:** Cesarean delivery, Race and ethnicity, Mode of delivery, Nulliparous, Healthcare disparity

## Abstract

**Background:**

Cesarean sections are the most common surgical procedure for women in the United States. Of the over 4 million births a year, one in three are now delivered in this manner and the risk adjusted prevalence rates appear to vary by race and ethnicity. However, data from individual studies provides limited or contradictory information on race and ethnicity as an independent predictor of delivery mode, precluding accurate generalizations. This study sought to assess the extent to which primary, unscheduled cesarean deliveries and their indications vary by race/ethnicity in one academic medical center.

**Methods:**

A retrospective, cross-sectional cohort study was conducted of 4,483 nulliparous women with term, singleton, and vertex presentation deliveries at a major academic medical center between 2006–2011. Cases with medical conditions, risk factors, or pregnancy complications that can contribute to increased cesarean risk or contraindicate vaginal birth were excluded. Multinomial logistic regression analysis was used to evaluate differences in delivery mode and caesarean indications among racial and ethnic groups.

**Results:**

The overall rate of cesarean delivery in our cohort was 16.7%. Compared to White women, Black and Asian women had higher rates of cesarean delivery than spontaneous vaginal delivery, (adjusted odds ratio {AOR}: 1.43; 95% CI: 1.07, 1.91, and AOR: 1.49; 95% CI: 1.02, 2.17, respectively). Black women were also more likely, compared to White women, to undergo cesarean for fetal distress and indications diagnosed in the first stage as compared to the second stage of labor.

**Conclusions:**

Racial and ethnic differences in delivery mode and indications for cesareans exist among low-risk nulliparas at our institution. These differences may be best explained by examining the variation in clinical decisions that indicate fetal distress and failure to progress at the hospital-level.

## Background

Cesarean sections are the most common surgical procedure for women in the United States. Of nearly four million births a year, one in three are delivered in this manner. The rate of cesarean delivery (CD) has increased more than 50% from 20.7% in 1996 to 32.8% in 2011 [1,2], without concurrent improvement in outcomes. Despite public health targets to reduce CD rates in low-risk primiparous women to 23.9% [3], rates remain high in this population [4] with evidence of racial and ethnic disparities. Some studies suggest a disproportionately higher rate among certain minorities, even when controlling for demographic, behavioral, medical, and institutional level factors [4-9]. Hypotheses suggested to explain these differences include variation in clinician decisions about labor management, hospital level characteristics, maternal preferences and risk tolerance, and/or unknown differences in labor patterns among subgroups of women [10-12]. However, the reported effect of race/ethnicity on primary cesarean rates is inconsistent among groups and between studies, possibly reflecting study design diversity, variation in the distributions of racial/ethnic groups’ prevalent in different geographic areas, and unreliable race/ethnicity measures. Furthermore, due to considerable intra-regional variation, [10,13], national data on primary CDs may not reflect local trends, substantiating the need to monitor rates at the individual hospital level [14]. Therefore, this study sought to assess the extent to which primary, unscheduled cesarean deliveries and their indications vary by race/ethnicity at a single tertiary-care academic center with a diverse urban and suburban population.

## Methods

After approval by the University of Massachusetts Medical School Institutional Review Board, we conducted a retrospective, cohort study using a subset of data exported from the University of Massachusetts Memorial Medical Center (UMMMC) Labor and Delivery electronic medical record (EMR) database. The database contains intrapartum and birth information prospectively collected by physicians and nurses for the clinical record. All women who delivered at UMMMC over a five-year period between April 1^st^, 2006 and March 31^st^, 2011 were considered for inclusion; thus data pertaining to 20,649 deliveries was initially accessed for analysis. Only nulliparous women who labored or attempted labor at term (37–41 weeks), with singleton, and vertex presentation deliveries were included. Cases with medical conditions, risk factors, or pregnancy complications that may contribute to increased cesarean risk or contraindicate vaginal birth were excluded; these included: gestational and pregestational diabetes, intrauterine growth restriction, vaginal bleeding, renal disease, HIV positive status, cardiac disease, trauma, uterine abnormality, lupus, chronic hypertension, pregnancy-induced hypertension (gestational hypertension, preeclampsia and eclampsia), placenta or vasa previa, previous myomectomy, active herpes infection, history of substance abuse, and absence of prenatal care [15,16]. Scheduled cesareans and non-live births were excluded, as well as cases with unspecified race/ethnicity or missing data on BMI. Data from primary patient records were consulted in cases where data discrepancies needed to be resolved or data on CD indication was missing.

The result was a study dataset of 4,483 low-risk nulliparous women with term, singleton, vertex presentation births who experienced spontaneous labor or underwent induction of labor, and were delivered by one of three modes: spontaneous vaginal delivery (SVD), operative vaginal delivery (OVD, including either forceps or vacuum assisted deliveries) or unplanned cesarean delivery (CD). Mode of delivery was the primary outcome for the analysis. The primary predictive variable was maternal race/ethnicity. This variable was based on self-reported versus attributed race/ethnicity information obtained from the patient record and grouped into four mutually exclusive categories: non-Hispanic Asian, non-Hispanic Black, Hispanic, or non-Hispanic White (henceforth referred to as Asian, Black, Hispanic, and White). More detailed race and ethnicity data were not available in the EMR.

Consideration was given to the following potential confounding variables, identified *a priori*: maternal age, body mass index (BMI), neonate size, and primary language. Maternal age was classified into the following groups: ≤19, 20- ≤ 24, 25- ≤ 29, 30- ≤ 34, and ≥ 35. Maternal BMI (kg/m^2^) was calculated based on self-report prepregnancy weight and height and grouped as follows: <18.5 (underweight), 18.5- ≤ 24.9 (normal weight), 25 - < 29.9 (overweight), and ≥ 30 (obese). The gestational age and birth weight of neonates were used to group infants into three classifications of size: small for gestational age (SGA), appropriate for gestational age (AGA), and large for gestational age (LGA). Neonates were considered to be SGA and LGA respectively if birth weights were <10^th^[17] and ≥90^th^[18] percentiles of 1999–2000 U.S. national reference data [19] for singletons, accounting for gestational age and gender [20]. Gestational age at delivery was based on best dates for estimated date of delivery as per clinician evaluation and as recorded in the EMR. The following primary languages: English, Spanish, Vietnamese, Portuguese, Laotian and ‘other’, were grouped as English or non-English speaking, respectively. For the purposes of our analyses, cases were grouped into one of three indication categories, all of which were designated during the intrapartum period: *first stage* (failed induction, prolonged latent phase, secondary arrest of dilation, prolonged active phase); *second stage* (arrest of descent, failure of descent, protracted descent, failed vacuum); and *fetal distress* (non-reassuring fetal heart tracing/intrapartum fetal distress, cord prolapse, placental abruption). If a woman had more than one indication for CD, only the primary indication, designated by the delivering physician, was retained for analyses. The delivering physician was either a resident, attending, or community-based provider.

Descriptive statistics were calculated for all study variables. Bivariate analyses using chi-squared test (*X*^2^) of independence and Fisher’s exact tests were conducted to identify variables associated with delivery mode. Variables whose unadjusted relationship with delivery mode was significant, at *p* < 0.5, were retained in the multivariate model. Multinomial logistic regression, using a stepwise approach, was then performed with the entire cohort for mode of delivery, with spontaneous vaginal delivery (SVD) acting as the reference category. The following independent variables were considered categorical in the model: race/ethnicity, maternal age, neonate size, and BMI. The reference racial/ethnic group was White (the majority group); the reference maternal age group was 25 - ≤ 29 years; the reference infant size was AGA; and the reference BMI category was the normal (BMI 18.5- ≤ 24.9) group. A second multinomial logistic regression was performed for CD indication, among cesarean deliveries only, with second stage indications acting as the reference category. The independent variable in this model was race/ethnicity, with White women, and AGA infant size acting as the reference groups. In a post-hoc analysis conducted to maximize statistical efficiency by ensuring adequate cell counts in clinically meaningful categories, first and second stage indications were combined and compared with fetal distress. First and second stage indications together essentially represent a clinical category of “failure to progress” or labor dystocia. The Wald (*X*^2^) statistic, adjusted odds ratio (AOR), and 95% confidence interval (CI) for the AOR were determined for both models. SAS [21] and SPSS [22] statistical analysis software were used for data management and statistical analyses.

**Table 1:** Patient characteristics by race and ethnicity

**Characteristic**	**Total**	**White**	**Black**	**Asian**	**Hispanic**
	**(n = 4,483)**	**(n = 3,168)**	**(n = 344)**	**(n = 204)**	**(n = 767)**
	**n/(%)**	**n/(%)**	**n/(%)**	**n/(%)**	**n/(%)**
Maternal age (yr)					
0- ≤ 19	43 (12.5)	345 (10.9)	43 (12.5)	7 (3.4)	311 (40.5)
20- ≤ 24	112 (32.6)	688 (21.7)	112 (32.6)	29 (14.2)	285 (37.2)
25- ≤ 29	124 (36.0)	987 (31.2)	124 (36.0)	72 (35.3)	108 (14.1)
30- ≤ 34	48 (14.0)	803 (25.3)	48 (14.0)	72 (35.3)	47 (6.1)
≥35	17 (4.9)	345 (10.9)	17 (4.9)	24 (11.8)	16 (2.1)
Body mass index					
Underweight	199 (4.4)	118 (3.7)	10 (2.9)	24 (11.8)	47 (6.1)
Normal weight	2,547(56.8)	1,790 (56.5)	184 (53.5)	152 (74.5)	421 (54.9)
Overweight	1,043 (23.3)	754 (23.8)	92 (26.7)	18 (8.8)	179 (23.3)
Obese	694 (15.5)	506 (16.0)	58 (16.9)	10 (4.9)	120 (15.6)
Neonate size					
SGA	410 (9.1)	238 (7.5)	34 (9.9)	39 (19.1)	99 (12.9)
AGA	3,766 (84.0)	2,674 (84.4)	296 (86.0)	159 (77.9)	637 (83.1)
LGA	307 (6.8)	256 (8.1)	14 (4.1)	6 (2.9)	31 (4.0)
Delivery mode					
SVD	3,322 (74.1)	2,333 (73.6)	251 (73.0)	136 (66.7)	602 (78.5)
OVD	411 (9.2)	300 (9.5)	23 (6.7)	28 (13.7)	60 (7.8)
ICD	750 (16.7)	535 (16.9)	70 (20.3)	40 (19.6)	105 (13.7)

*SGA* small for gestational age, *AGA* appropriate for gestation age, *LGA* large for gestational age.

*SVD* spontaneous vaginal delivery, *OVD* operative vaginal delivery, *ICD* intrapartum cesarean delivery.

## Results

**Figure 1:**
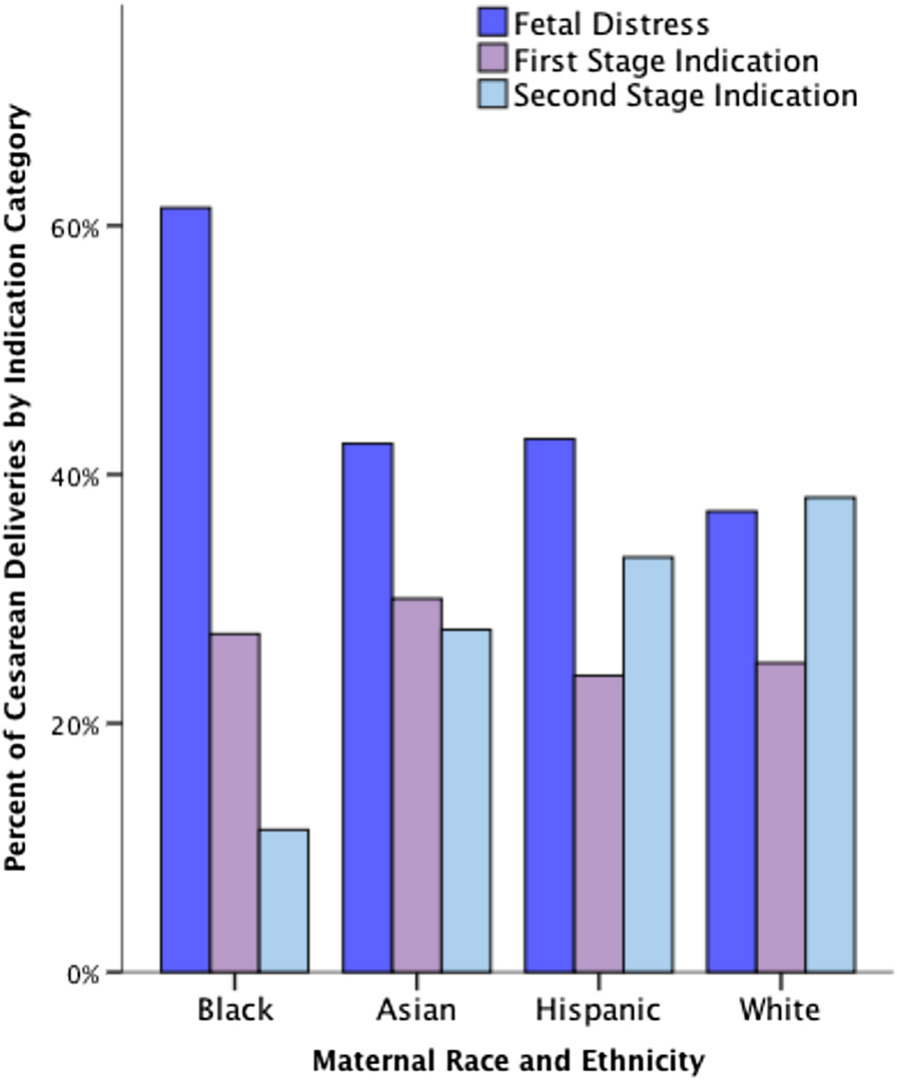
Cesarean delivery indications by race and ethnicity.

In the five-year study period there were 20,649 deliveries of which 4,483 (21.7%) met criteria for inclusion. Included women had singleton, vertex presentation, full-term deliveries and were without medical conditions, pregnancy complications or pre-labor CD indications as previously outlined. Subjects were thus considered ‘low-risk’ for CD. There were no significant differences between the overall population and the study cohort with regard to maternal age, race/ethnicity, BMI, and birth weight. Table 1 presents descriptive data pertaining to all study variables stratified by race and ethnicity. Maternal age ranged from 14–47 years (mean, 26.2 ± 5.9) and the mean BMI was 25 kg/m^2^ ± 5.5. The racial/ethnic composition of the sample was 70.7% White, 17.1% Hispanic, 7.7% Black, and 4.6% Asian. Overall, 74.1% had a SVD, 16.7% had a CD and 9.2% had an OVD. The indication category for CD was highest for fetal distress (40.4%), followed by indications assigned during the second stage of labor (34.4%), and then by indications assigned during the first stage of labor (25.2%). Non-reassuring fetal heart tracings or intrapartum fetal distress constituted 98.3% of the fetal distress indication category. Arrest of dilation and prolonged labor made up 87.1% of the first stage indication category. Failure to descend, arrest of descent, and protracted descent represented 78.4% of the second stage indication category. An unadjusted analysis of CD indications by race and ethnicity (Figure 1), revealed that among Black women, 61% of CD were for fetal distress, compared with a rate of 37 to 43 percent for White, Asian, and Hispanic women (p < 0.01). There were significant effects of maternal age, BMI, and neonate size on mode of delivery. As expected, compared with women who had a SVD, older women (≥ 35 years of age), overweight and obese women, and women who delivered LGA neonates were more likely to have a CD. There were no significant differences in mode of delivery by English and non-English speaking women.

Table 2 shows the results of the multinomial logistic regression model for mode of delivery. The outcome suggests that compared with White women, Black women had higher rates of primary CD compared to SVD, after controlling for significant confounding variables, (AOR 1.43; 95% CI: 1.07, 1.91, *p* < 0.01). Asian women also had higher rates of primary CD compared to SVD, (AOR 1.49; 95% CI: 1.02, 2.17, *p* < 0.04). There were no statistically significant differences in delivery mode between White women and Hispanic women or between OVD and SVD among all women, despite a trend suggesting higher OVD among Asian women.

**Table 2:** Multinomial logistic regression results for mode of delivery

**Characteristic**	**SVD**	**ICD**		**OVD**	
	**n (%)**	**n (%)**	**AOR (95% CI)**	**n (%)**	**AOR (95% CI)**
Race/ethnicity					
White	2333 (73.6)	535 (16.9)	Reference	300 (9.5)	Reference
Black	251 (73.0)	70 (20.3)	**1.43 (1.07-1.91)**	23 (6.7)	0.80 (0.51-1.23)
Asian	136 (66.7)	40 (19.6)	**1.49 (1.02-2.17)**	28 (13.7)	1.40 (0.91-2.15)
Hispanic	602 (78.5)	105 (13.7)	1.14 (0.89-1.47)	60 (7.8)	1.03 (0.75-1.42)

SVD is reference group.

Row percent displayed.

Model adjusted for maternal age, BMI, and neonate size.

*SVD* spontaneous vaginal delivery, *ICD* intrapartum cesarean delivery, *OVD* operative vaginal delivery.

Bold values reflect p < 0.05.

Table 3 shows the results of the multinomial logistic regression model for CD indications. Compared to White women, Black women were significantly more likely to undergo CD for fetal distress (AOR: 5.28; 95% CI: 2.36, 11.81). They were also more likely to undergo CD for indications diagnosed in the first stage of labor (AOR: 3.59; 95% CI: 1.50, 8.63), as compared to indications diagnosed in the second stage of labor, after controlling for neonate size. Table 4 summarizes the results of the post-hoc binary regression, which shows that compared to White women, Black women were more likely to undergo fetal distress (AOR: 2.60, 95% CI: 1.52, 4.45), as compared to all other indications. There were no further significant differences in indication categories by race/ethnicity in the multinomial or binary regression for CD indications.

**Table 3:** Summary of multinomial logistic regression analysis for race and ethnicity associated with cesarean indication categories (n = 750)

**Characteristic**	**Second stage**	**First stage**		**Fetal distress**	
	**n (%)**	**n (%)**	**AOR (95% CI)**	**n (%)**	**AOR (95% CI)**
**Total/n (%)**	**258 (34.4)**	**190 (25.2)**		**302 (40.4)**	
Race/ethnicity					
White	204 (38.1)	134 (24.8)	Reference	197 (37.0)	Reference
Black	8 (11.4)	19 (27.1)	**3.59 (1.50, 8.63)**	43 (61.4)	**5.28 (2.36,11.81)**
Asian	11(27.5)	12 (30.0)	2.35 (0.96, 5.76)	17 (42.5)	1.28 (0.54, 3.05)
Hispanic	35 (33.3)	25 (23.8)	0.91 (0.49, 1.69)	45 (42.9)	0.79 (0.45, 1.39)

Second Stage is reference group.

Row percent displayed.

Model adjusted for maternal age, BMI, and neonate size,

Bold values reflect p < 0.05.

**Table 4:** Summary of binary logistic regression analysis for race and ethnicity associated with cesarean indications, odds of fetal distress vs. failure to progress

**Race/ethnicity**	***B***	**SE**	**Wald**	**AOR (95% CI)**
Black vs. White	.931	.267	12.14	**2.60 (1.52, 4.45)**
Asian vs. White	-.204	.364	.313	0.89 (0.43, 1.85)
Hispanic vs. White	0.92	.226	.164	0.83 (0.51, 1.36)

Model adjusted for maternal age, BMI, and neonate size.

Bold values reflect p < 0.05.

## Discussion

The study offers current data that demonstrates that racial and ethnic differences in mode of delivery and CD indications exist in a sub-population of low-risk women with access to labor and delivery services in the same healthcare system. We observed a higher rate of primary, unscheduled, intrapartum CD among Black and Asian women relative to their White counterparts, after controlling for demographic and medical risk factors. We also observed that Black women were more likely to undergo a CD for fetal distress and failure to progress compared to White women.

Our finding of higher risk of CD for fetal distress among Black women is consistent with those of two studies from California [4,23]. Getahun, et al.’s study, based on data from Kaiser Permanente Southern California over 17 years, found Black women had higher rates of more subjective indications such as fetal distress and ‘other indications’, compared with White, Hispanic, and Asian/Pacific Islander women. However, the study lacked data on BMI and the results were not stratified by parity. A similar study by Washington and colleagues, based on data from University of California San Francisco between 1990 and 2008, also found higher rates of non-reassuring fetal heart tracing among term, primiparous Black women compared to their White counterparts, adjusted for BMI among other potentially confounding variables.

Additional comparisons with previous studies are imprecise. Exact definitions of race and ethnicity and ‘low-risk’ births are not universally accepted nor consistently applied in this field. Accordingly, different categories of race and ethnicity are used and there is lack of consensus on the method for risk-adjustment. Furthermore, the diverse distributions of racial and ethnic sub-groups prevalent within different institutional catchment areas complicate the comparison of studies. Bryant and colleagues suggests that the strength of the existing evidence for racial/ethnic disparities in primary CD supports socio-cultural circumstances and shortfalls in medical care [24], particularly among primary, intrapartum CDs as they are more consistently impacted by provider dependent labor management decisions. The known subjectivity in the assessment of fetal distress using electronic fetal monitoring [25]; supports the potential of variation in decision making that results in lower thresholds for diagnosing fetal distress and recommending a cesarean among certain racial/ethnic groups. Thus, further examination into clinician decision-making about CD indications together with women’s influence and involvement in these decisions may help explain the observed differences. Examination of variations in fetal tolerance for labor and the timing of CD in relation to labor progress may also be warranted.

What is novel about this study is that the data pertains to a considerably low-risk population of nulliparous women who were candidates for vaginal delivery and yet who underwent unscheduled, intrapartum primary cesarean sections. Second, multinomial logistic regression analysis, that employed three modes of delivery as the primary outcome, distinguished the potential influence of race/ethnicity on operative and spontaneous vaginal deliveries. The strengths of our study include a large sample of relatively diverse women derived from a clinical database. Use of a clinical database allowed the isolation of a representative sample of women at low-risk for CS through use of restrictive clinical criteria, aiding in the evaluation of the influence of the non-clinical factor race/ethnicity. Prospectively collected medical record data with physician-documented indications for labor was used, as opposed to birth certificate data, which is known to be non-specific and contain numerous inaccuracies [26-28].

Yet, several limitations deserve consideration. First, the data was collected from a single academic medical center in Massachusetts, and thus may not be generalizable to populations with different demographic, regional characteristics, and clinical practice patterns. However, as reported by the Department of Health and Human Services Action Plan to Reduce Racial and Ethnic Disparities [29], there is a demand for local data that is reflective of the unique population makeup of an institution’s catchment area. Accordingly, our findings may be community-specific, particularly given the large foreign-born West African immigrant population in our region. Results from a study of all births from 1998 to 2006 in Massachusetts indicate that the primary CD rates are almost twice as high for Black non-Hispanic mothers than for White non-Hispanic mothers, determined almost entirely by the foreign born cohort of Black non-Hispanic mothers [30]. Future studies should consider nativity and more detailed data on ethnicity.

Second, unmeasured confounding bias may contribute to the observed differences. Most notably our data source lacked indicators of socioeconomic status (SES) such as education and income level, factors known to impact health outcomes. Yet, access to care is one of the most common pathways by which low SES is thought to act on health care utilization and the data used in this study was obtained from medical records of UMass Memorial Health Care patients, all of who were presumed to have equivalent access to this facility; therefore lack of access to delivery services is not a likely determinant in our study. Results of studies that have included indicators of SES, have demonstrated that SES does not explain away the differences of race and ethnicity on mode of delivery [5,6,31,32]. Nonetheless, the study had limited ability to determine how SES might have played a role in the observed differences. Noteworthy, patients were a mix of public and private pay.

Third, women were included in the study if they experienced spontaneous labor or underwent induction of labor. Studies have shown that induction of labor increases the risk of caesarean delivery [33,34]. However, in controlling for maternal age [35] and BMI [36], we reduced the likelihood of a confounding effect as these variables are associated with induction of labor. Further, a recent study found that significant associations between CD and race persisted after adjustment for induction in nulliparous women at term [37]. Therefore, we believe that our associations would remain. Finally, a minor limitation is the use of self-reported pre-pregnancy weight, which is reported to be underestimated compared to weight measured at the first prenatal visit [38].

## Conclusions

Racial and ethnic differences in delivery mode and indications for cesareans exist at our institution among a low-risk population of women. Future studies are needed to explain the increased risk among Black and Asian women and the apparent variation in clinical decisions that indicate fetal distress and failure to progress among Black women. The National Quality Forum’s Perinatal Care guidelines [39], for assessing the number of low-risk first birth women delivered by cesareans, does not require stratification by race and ethnicity, despite acknowledgement of racial disparities. Yet, hospitals looking to improve consistency and equality may want to analyze their rates, by locally prevalent race and ethnicity patient populations. Variability in hospital rates of cesareans is of public health significance, and hospital specific data that captures the unique racial/ethnic distribution of a population may help better explain the variation in low-risk cesarean rates and promote standard application of clinical guidelines for intrapartum care.

## Abbreviations

CD: Cesarean delivery; ICD: Intrapartum caesarean delivery; EMR: Electronic medical record; SVD: Spontaneous vaginal delivery; OVD: Operative vaginal delivery; BMI: Body mass index; SGA: Small for gestational age; AGA: Appropriate for gestational age; LGA: Large for gestational age; SES: Socioeconomic status.

## Competing interests

The authors declare that they have no competing interests.

## Authors’ contributions

JKE conceived of the study, participated in its design, and drafted the manuscript. RY assisted with data collection, performed chart reviews, and helped with revision of the manuscript. XL performed the statistical analysis. TMS participated in the design and coordination of the study and helped with revision of the manuscript. All authors read and approved the final manuscript.

## Authors’ information

JKE is Assistant Professor at William F. Connell School of Nursing, Boston College. RY is a Resident Physician in the Department of Obstetrics and Gynecology, University of Massachusetts Medical School/UMass Memorial Medical Center. XL is a Statistical Analyst in the Department of Obstetrics & Gynecology, University of Massachusetts Medical School. TAMS is Associate Professor of Obstetrics and Gynecology and Pediatrics and OB/GYN Research Division Director in the Department of Obstetrics and Gynecology, University of Massachusetts Medical School/UMass Memorial Medical Center.

## Pre-publication history

The pre-publication history for this paper can be accessed here:

http://www.biomedcentral.com/1471-2393/13/168/prepub
